# Is Montreal Cognitive Assessment a valuable test for the differentiation of Alzheimer's disease, frontotemporal dementia, dementia with Lewy body, and vascular dementia?

**DOI:** 10.1590/1980-5764-DN-2023-0124

**Published:** 2024-08-26

**Authors:** Fatemeh Afrashteh, Mostafa Almasi-Dooghaee, Naser Kamyari, Rayan Rajabi, Hamid Reza Baradaran

**Affiliations:** 1 Iran University of Medical Sciences, School of Medicine, Tehran, Iran.; 2 Iran University of Medical Sciences, Department of Neurology, Firoozgar Clinical Research Development Center (FCRDC), Tehran, Iran.; 3 Abadan University of Medical Sciences, Department of Biostatistics and Epidemiology, School of Health, Abadan, Iran.; 4 Iran University of Medical Sciences, Department of Epidemiology, School of Public Health, Tehran, Iran.; 5 University of Aberdeen, Ageing Clinical and Experimental Research Team, Institute of Applied Health Sciences, Aberdeen, UK.

**Keywords:** Mental Status and Dementia Tests, Alzheimer Disease, Frontotemporal Dementia, Lewy Body Disease, Dementia, Vascular, Testes de Estado Mental e Demência, Doença de Alzheimer, Demência Frontotemporal, Doença por Corpos de Lewy, Demência Vascular

## Abstract

Dementia is one of the growing diseases in the world and has different types based on its definition. The Montreal Cognitive Assessment (MoCA) test has been employed to screen patients with dementia, cognitive impairment, and disruption of daily activities.

**Objective:**

This study examined the diagnostic value of the total MoCA score and its subscores in differentiating Alzheimer's disease (AD), frontotemporal dementia (FTD), dementia with Lewy body (DLB), and vascular dementia (VaD).

**Methods:**

A total of 241 patients (AD=110, FTD=90, DLB=28, and VaD=13) and 59 healthy persons, who were referred to a dementia clinic with memory impairment in Firoozgar Hospital, were included in this study. MoCA tests were performed in all patients and normal persons.

**Results:**

By using the receiver operating characteristic (ROC) curve and measuring the area under the curve (AUC) for the total MoCA score in each group, AUC was 0.616, 0.681, 0.6117, and 0.583 for differentiating AD, FTD, DLB, and VaD patients, respectively. Among the groups, just the VaD group showed no significant usefulness in using the total MoCA score to differentiate it. To compare MoCA subscores, AD patients had higher scores in digit span, literal fluency, and abstraction but lower delayed recall scores compared with FTD patients.

**Conclusion:**

The total MoCA score and its subscores could not differentiate people with different types of dementia in the setting of screening.

## RESUMO

A demência é uma das doenças que mais cresce no mundo e possui diferentes tipos de acordo com sua definição. O teste de Avaliação Cognitiva de Montreal (*Montreal Cognitive Assessment* – MoCA) tem sido empregado para a triagem de pacientes com demência, comprometimento cognitivo e perturbação das atividades diárias.

**Objetivo:**

Este estudo examinou o valor diagnóstico da pontuação total do MoCA e seus subescores na diferenciação entre doença de Alzheimer (DA), demência frontotempotal (DFT), demência com corpos de Lewy (DCL) e demência vascular (DV).

**Métodos:**

Este estudo incluiu 241 pacientes (DA=110, DFT=90, DCL=28 e DV=13) e 59 pessoas saudáveis, encaminhados à clínica de demência com comprometimento de memória no Hospital Firoozgar. O teste MoCA foi realizado em todos os pacientes e pessoas normais.

**Resultados:**

Usando a curva característica de operação do receptor (ROC) e medindo a área sob a curva (AUC) para a pontuação total do MoCA em cada grupo, a AUC foi de 0,616, 0,681, 0,6117 e 0,583 para diferenciar pacientes com DA, DFT, DCL e DV, respectivamente. Entre os grupos, apenas o grupo DV não mostrou utilidade significativa no uso do escore total do MoCA para diferenciá-lo. Para comparar os subescores do MoCA, os pacientes com DA tiveram pontuações mais altas em amplitude de dígitos, fluência verbal e abstração, mas menor pontuação de recordação tardia em comparação com pacientes com DFT.

**Conclusão:**

A pontuação total do MoCA e seus subescores não conseguiram diferenciar pessoas com diferentes tipos de demência no contexto da triagem.

## INTRODUCTION

Dementia is defined as a significant reduction in cognitive and daily functions which encompasses a wide range of conditions^1^. Alzheimer's disease (AD), which is the most common cause of dementia, is a neurodegenerative disease and causes progressive cognition decline^2^. The second cause of dementia, including 15% of people with dementia, is vascular dementia (VaD) which develops after brain stroke, in 15–30% of patients in 3 months and in 20–25% of patients more than 3 months after stroke^3,4^. Frontotemporal dementia (FTD), which is a common cause of young-onset dementia, is a group of diseases indicated by atrophy in the frontal and temporal lobes^5,6^. Dementia with Lewy body (DLB), which is the second cause of neurodegenerative dementia after AD, is observed in 4–8% of dementia cases and is presented by fluctuation in cognition impairment, parkinsonism, and visual hallucination^7,8^.

Although each type of dementia has its own criteria mostly based on imaging and clinical features, using screening tools to diagnose each type and consider the appropriate management of the patients could be helpful. The Montreal Cognitive Assessment (MoCA) is used as a new screening tool for detecting healthy patients who have mild cognitive impairment (MCI) with an acceptable specificity (87%) and higher sensitivity (90%) than the Mini-Mental State Exam (MMSE), which is the most common screening tool for this purpose^9,10^. MoCA is an available and easy-to-use tool that could be performed to screen patients with dementia. If a patient (a person) obtains a score from 0 to 30, those with a score of 26 or higher are considered healthy, and lower scores are considered to have MCI, which is the condition between a healthy state and dementia, or other dementia types. These fields of cognition are evaluated by MoCA: visuospatial/executive function, memory, attention, abstraction, language, and orientation^10^. Each subscore in MoCA is representative of different damage in the brain parts that are damaged with different patterns in the types of dementia. As MoCA examines various parts of cognition, using only the total MoCA score to differentiate types of dementia that have different physiopathology and symptoms is not enough.

According to previous reports, the prevalence of dementia will increase to more than 115 million by 2050, which was 36 million people in 2010^11^. The rising prevalence of dementia in the general population increases the need for having a valid screening tool for physicians. In addition, when dementia worsens, the more the exorbitant costs of the society will increase, so it is necessary to identify patients with dementia at early stages^12^.

Some studies have evaluated the role of MoCA in the differentiation of some types of dementia, and the results from these studies give us the clue that MoCA subscores and total scores could be useful tools to reach this purpose^13–16^. However, including all types of dementia with a larger sample size was needed. In this study, we evaluated the sensitivity and specificity of the total MoCA score in the differentiation of AD, FTD, DLB, and VaD from each other. Also, we compared the subscores of MoCA between these diseases and the healthy group to investigate whether MoCA could be used as a valuable screening test to help differentiate dementia types from each other and healthy persons.

## METHODS

After granting ethical approval from the ethics committee of Iran University of Medical Sciences (number IR.IUMS.FMD.REC.1400.254), all patients who were referred to the dementia clinic in Firoozgar Hospital (Tehran, Iran) from March 2017 to March 2022 if they had the following criteria were included in our study:

- Written informed consent to participate in this study from each person or their family (or their health care) if the patient could not;
- At least 6 years of education;
- Having mastery of Persian language;
- Having brain MRI.

The exclusion criteria were as follows:

- Having physical limitation or severe visual and hearing impairment that could interfere with cognitive testing;
- Having a history of neurological disease (such as epilepsy, head trauma, stroke, metabolic disease, etc.) that could cause disturbances in cognitive testing.

First, the Persian version of the MoCA test^17^ was performed by an expert academic neurologist, and the scores were recorded for each participant. To correct for the effect of education, one point is added to the total score of participants with less than 12 years of education. The following scores were calculated for each participant alongside MoCA subscores:

- The memory index score (MIS)^18^: Adding up the scores in the following order: three scores for words that are remembered without guidance. Two points for words recalled with a hint. A score for words that are remembered with more guidance and giving the person options;
- Literal fluency: The number of words that are started with a specific alphabet that the patient could say in one minute ("m" in Persian);
- Semantic fluency: The number of animal names that the patient could say in one minute.

After performing the MoCA test, another neurologist, who was unaware of the MoCA results, visited and examined the patients, and according to the below criteria, each patient was diagnosed with any of these dementia: AD, FTD, DLB, and VaD. AD diagnosis was based on the NINCDS-ADRDA criteria^19^. FTD criteria are different for each type of FTD: bvFTD, nfFTD, svFTD, and progressive supranuclear palsy FTD (PSP-FTD) are described previously in the studies^20,21^. Based on the Consortium for DLB Diagnostic Criteria, DLB was considered for those who had these criteria^22^. DSM-V criteria were used for VaD diagnosis^23^.

The patients who had normal examinations and interviews and obtained a total MoCA score≥26 were considered healthy.

The quantitative data were reported as mean ± standard deviation (with a 95% confidence coefficient), and the qualitative data were also described as frequency percentages or numbers. The receiver operating characteristic (ROC) curve was used to detect the sensitivity and specificity of the total score of the MoCA test, the optimal cut-off for each diagnosis, and the area under the curve (AUC). Analytical analysis was performed using one-way ANOVA and the post-hoc Tukey test. p<0.05 was considered statistically significant.

## RESULTS

In this cross-sectional study, 300 individuals including 179 males (58.1%) and 121 females (39.3%) participated. The mean age of the participants was 69.9±9.5 years. Among these 300 participants, 59 (19.2%) healthy participants, 110 (35.7%) AD, 90 (29.2%) FTD, 28 (9.1%) DLB, and 13 (4.2%) VaD were diagnosed, respectively. Among FTD patients, 55 bvFTD, 11 nfFTD, 14 svFTD, and 4 PSP-FTD were diagnosed, but six patients could not be placed under any FTD category.

The mean year of education was 12.9±5.8 years. The mean duration of symptoms at the time of examination of patients was 25.8±25.9 months. The duration of symptoms was not different between dementia groups. The demographic data of the participants are shown in Table 1.

**Table 1:** The demographic data of the participants.

	AD (n=110)	FTH (n=90)	DBL (n=28)	VaD (n=13)	Healthy controls (n=59)	All participants (n=300)
Age (years)	74.09±7.163	68.46±8.538	74.25±5.282	69.69±6.277	62.53±11.886	69.95±9.566
Female/male (n)	45/65	36/54	9/19	1/12	30/29	121/179
Education (years)	12.93±7.469	12.07±4.593	11±5.200	12.00±3.916	15.48±3.733	12.94±5.859
Duration of symptoms (month)	25.88±19.424	27.15±26.030	24.00±19.542	16.09±14.046	27.55±45.482	25.86±25.928

Abbreviations: AD, Alzheimer's disease; FTD, frontotemporal dementia; DLB, dementia with Lewy body; VaD, vascular dementia.

Memory loss was the most common chief complaint of the patients in all groups except FTD which was commonly presented by behavioral change and memory loss was the second cause in this group. The second cause of chief complaint was different in these groups: behavioral change in AD and VaD, visuospatial problems in DLB, and other complaints in the normal group.

The patients in the AD group had higher age than the healthy group (p<0.001) and FTD group (p<0.001). Like AD, the DLB group had a higher age than the healthy group (p<0.001) and FTD (p=0.011) group. The patients in the AD, FTD, and DLB groups experienced lower years of education than healthy ones (p<0.001).

The total MoCA score measured 16.2±7.5 in all participants, 27.2±1.3 in the healthy group, 14.1±5.2 in the AD group, 12.8± 6.5 in the FTD group, 13.5±6.2 in the DLB group, and 13.9±5.1 in the VaD group. However, the total MoCA score was not statistically significantly different in the dementia groups, but the healthy persons had a higher total MoCA score than the dementia groups (p<0.001 for all groups). The means of each MoCA item in total participants and each group are shown in Table 2.

**Table 2:** The mean of Montreal Cognitive Assessment items in total patients and each group.

Item	Total patients (n=300)	Normal (n=59)	AD (n=110)	FTH (n=96)	DLB (n=28)	VaD (n=13)
Trail-making score	0.280±0.451	0.93±0.25	0.15±0.35	0.12±0.33	0.07±0.26	0.08±0.27
Cube copy score	0.383±0.487	0.93±0.253	0.25±0.437	0.25±0.44	0.25±0.44	0.15±0.37
Clock drawing score	1.860±0.950	2.95±0.22	1.65±0.92	1.53±0.80	1.57±0.88	1.62±0.87
Naming score	2.460±0.750	2.93±0.25	2.41±0.76	2.31±0.80	2.25±0.75	2.23±1.01
Registration score	3.073±1.497	4.47±0.68	2.97±1.31	2.47±1.54	2.61±1.52	2.69±1.44
Digit span score	1.250±0.780	1.78±0.46	1.27±0.70	0.90±0.79	1.18±0.86	1.23±1.01
Vigilance score	0.506±0.513	0.90±0.30	0.46±0.52	0.36±0.48	0.43±0.57	0.23±0.44
Serial seven score	1.763±1.154	2.88±0.38	1.56±1.05	1.42±1.21	1.36±1.03	1.62±1.12
Repetition score	0.856±0.819	1.68±0.51	0.73±0.78	0.52±0.70	0.75±0.80	0.77±0.73
Verbal fluency score	0.316±0.465	0.81±0.39	0.23±0.42	0.16±0.37	0.25±0.44	0.00±0.00
Literal fluency score	7.635±5.320	13.29±4.04	7.05±4.21	5.28±4.98	7.07±4.85	4.23±3.70
Semantic fluency score	10.352±6.171	19.38±4.20	9.70±4.44	7.84±5.10	9.32±5.34	5.92±4.68
Memory index score	6.10±4.82	12.90±1.94	4.01±2.84	4.95±4.35	4.68±4.70	4.00±3.27
Abstraction score	0.77±0.85	1.73±0.55	0.69±0.81	0.36±0.60	0.36±0.68	0.92±0.86
Delayed recall score	1.25±1.67	3.78±1.02	0.37±0.81	0.85±1.35	0.86±1.35	0.31±0.63
Time orientation score	2.64±1.48	3.97±0.18	2.38±1.41	2.26±1.57	2.11±1.52	2.69±1.25
Place orientation score	1.65±0.58	2.00±0.00	1.60±0.53	1.51±0.72	1.54±0.69	1.77±0.44
Visuospatial/executive score	2.52±1.62	4.81±0.47	2.05±1.35	1.91±1.22	1.89±1.31	1.85±1.07
Attention score	3.50±1.86	5.47±0.80	3.30±1.74	2.69±1.97	2.96±1.93	3.08±1.98
Language score	1.18±1.10	2.53±0.68	0.95±0.99	0.69±0.83	1.00±1.05	0.77±0.73
Orientation score	4.30±1.86	5.97±0.18	3.98±1.65	3.77±2.09	3.64±2.06	4.46±1.51
Total MoCA score	16.26±7.60	27.25±1.36	14.12±5.27	12.84±6.53	13.57±6.28	13.92±5.09

Abbreviations: AD, Alzheimer's disease; FTD, frontotemporal dementia; DLB, dementia with Lewy body; VaD, vascular dementia; MoCA, Montreal Cognitive Assessment.

The healthy participants had higher scores than other groups in each item (p<0.05) except digit span and place orientation which were not statistically significantly different between the VaD group and healthy ones (p=0.099 and 0.662, respectively). However, AD patients showed higher digit span, literal fluency, and abstraction score than FTD patients (p=0.003, p=0.045, and p=0.010, respectively), but delayed recall score was lower in AD than FTD patients (p=0.010) (Table 3).

**Table 3:** The different scores in Montreal Cognitive Assessment items between dementia groups.

Group 1–Group 2	Item	Mean difference	p-value
AD-FTD	Digit span score	0.372	0.003
AD-FTD	Literal fluency score	0.636	0.045
AD-FTD	Abstraction score	0.324	0.010
FTD-AD	Delayed recall score	0.493	0.010

Abbreviations: AD, Alzheimer's disease; FTD, frontotemporal dementia.

**Figure 1:**
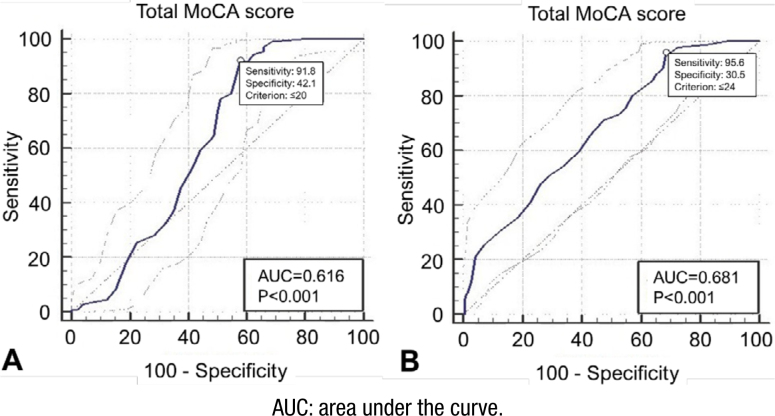
The receiver operating characteristic curve, the area under the receiver operating characteristic curve, sensitivity, specificity, and the optimal cut-off for differentiating Alzheimer's disease (A) and frontotemporal dementia (B).

**Figure 2:**
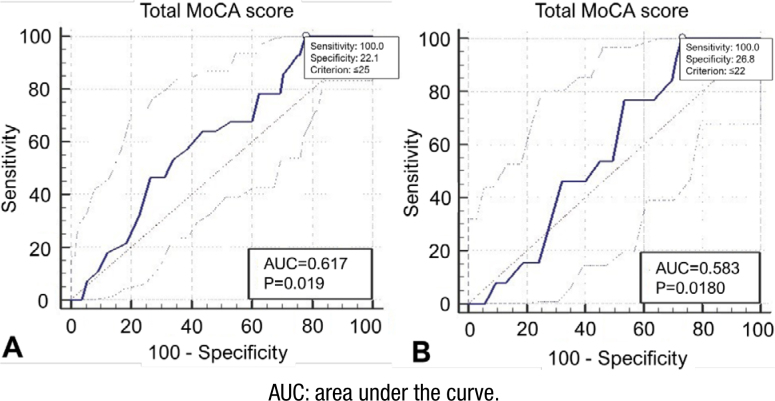
The receiver operating characteristic curve, the area under the the receiver operating characteristic curve, sensitivity, specificity, and the optimal cut-off for differentiating dementia with Lewy body (A) and vascular dementia (B).

ROC curve analysis was performed to obtain the accuracy (sensitivity and specificity) of the total MoCA score in differentiating each group. A total MoCA score ≤20 was detected as an optimal cut-off for differentiating AD patients from other conditions (sensitivity: 0.91, specificity: 0.42, p<0.001, AUC=0.61 (95%CI 0.55–0.67)). This optimal cut-off was ≤24 for FTD differentiation (sensitivity: 0.95, specificity: 0.30, p<0.001, AUC=0.681 (95%CI 0.62–0.73)). DLB showed higher cut-off and sensitivity than other dementia, with ≤25 as an optimal point (sensitivity: 1, specificity: 0.221, p=0.019, AUC=0.617 (95%CI 0.56–0.67)). Total MoCA score had no significant role in differentiating VaD patients from other patients (sensitivity: 1, specificity: 0.26, p=0.180, AUC=0.58 (95%CI 0.52–0.63)). ROC curves for all groups are shown in Figures 1 and 2. The adjusted ROCs (AROCs) were also performed by employing logistic regression for age, gender, and education; therefore, after adjustment, all AUC values increased these variables: 0.714 for AD (95%CI 0.659–0.764), 0.703 for FTD (95%CI 0.647–0.754), 0.703 for DLB (95%CI 0.655–0.761), and 0.751 (95%CI 0.698–0.799) for VaD.

## DISCUSSION

In this study, we measured the sensitivity and specificity of the total MoCA score to differentiate each type of dementia from other patients. Then, we compared the MoCA subscores in each group and healthy persons to determine which scores could be indicators of each dementia. In general, the total MoCA score and subscores could not differentiate types of dementia with a desirable sensitivity and specificity.

We evaluated that the total MoCA score is not very powerful in distinguishing AD from other dementia or healthy elderly is not with AUC=0.611. AUC showed a range of 0.87–0.99 in another study which is higher than what we measured^24^. This is because we included patients with other types of dementia alongside healthy persons, and this could affect the accuracy of the total MoCA score. Like AD, we found that the total MoCA is not much valuable to differentiate FTD patients from other patients, probably because we did not differentiate types of FTD to draw ROC because the number of patients was not enough. The total MoCA score showed a better in a study to differentiate bvFTD from AD and healthy persons^15^. We did not detect the validity of the total MoCA score to differentiate VaD from other patients, and this may be due to the low number of VaD in our study. In addition, although every subscore of normal persons was significantly higher than other groups, the place orientation and digit span score were intact in VaD. Unlike these results, the total MoCA score was useful in differentiating VaD patients in a study^25^. However, a mini-MoCA test that merges the clock drawing test, five-word delayed recall, and abstraction score, with high sensitivity and specificity, was suggested as a useful tool to screen cognitive impairment in stroke clinics^26^.

Overall, using only the total MoCA score is not enough to differentiate AD, FTD, and DLB from other patients or normal persons. For this purpose, the highest and lowest sensitivity values of the total MoCA score were 100% and 91.8% for DLB and AD, respectively, but the specificity was very low to differentiate these groups from each other. Today, the total MoCA score is an accessible and valid tool to screen patients with dementia in the general population, but a neurologist needs further investigations and imaging to diagnose each type of dementia and make a treatment plan.

However, the total MoCA score did not show an appropriate specificity to differentiate types of dementia; MoCA subscores could give us some clues that each person may fit into which categories. MoCA subscores have been evaluated in studies as solitary tasks or in MoCA tests to determine cognition profiles in types of dementia. The reason behind these evaluations comes from the evidence showing that each task could stimulate different cortex areas previously proved by using functional MRI imaging^27,28^.

The clock drawing test was not a helpful item to parse types of dementia. Formerly, the clock drawing test has been shown as a beneficial tool to screen healthy persons from those with dementia^29^. Visuospatial/executive impairment has been reported in both AD and Huntington's disease (HD), and the clock drawing test was shown to be destroyed in both diseases^30^. The errors in the clock drawing test were not the same in HD and AD; graphic difficulties were common in HD patients, but conceptual problems were particularly seen in AD patients and were related to the severity of AD. In another study about the qualitative errors in clock drawing test in AD and VaD, the later stages of VaD show more frequent graphic and conceptual errors but a lower frequency of spatial and/or planning errors than mild impairment in VaD^31^. These qualitative errors were not different in different severities of AD, unlike the previous study.

Subscores of DLB patients did not show any difference compared with subscores of other groups including AD in our study. Contrary to our study, lower scores in visuospatial/executive function, naming, and language were more suggestive of DLB than AD in the study^32^.

We found that AD patients had better performance in digit span, literal fluency, and abstraction but worse performance in delayed recall than FTD patients. We suggest that a delayed recall score may be useful to differentiate AD patients from other dementia like FTD. Repetition score in AD patients score was also impaired in our results.

MoCA items are previously used as sensitive and specific tests to differentiate FTD patients from related disorders such as bvFTD, nfFTD, primary progressive aphasia (nvPPA), semantic variant primary progressive aphasia (svPPA), PSP, and corticobasal syndrome (CBS)^13^. They found that the MoCA trail-making test had low sensitivity and high specificity compared with the gold standard test named trail-making test B. Rivermead delayed recall was used as a gold standard test of delayed recall test in MoCA, and they found that the delayed recall test in MoCA has high sensitivity but low specificity to differentiate these disorders. MoCA fluency test had high sensitivity and high specificity compared with the gold standard test named F/A/S Fluency test. Clock test and immediate recall scores in the MoCA test were also correlated with the full clock test and Rivermead immediate recall. In line with our study, these data suggested that most MoCA items, except trail making test, are good representatives for full tests of each cognitive domain in FTD and related disorders.

A study found that FTD patients had better performance in total MoCA score, serial seven, delayed recall, and orientation test, but vigilance and attention were the items that AD patients showed better performance^14^. According to impaired repetition and sentence processing in AD patients, a repetition test using six types of sentences to repeat was impaired in a study compared with normal persons^33^.

MIS scores of our participants were not significantly different between the groups. Despite our results, MIS, a score measured by physicians in delayed recall tasks during performing MoCA, displayed as a predictor of AD conversion from MCI^18^.

The diagnostic tools for differentiating the types of dementia to screen patients are limited. The types of imaging including MRI are not recommended preferred choices for screening dementia in the general population. As a less expensive technique, transcranial sonography was used previously to differentiate the types of dementia including AD, FTD, DLB, and VaD^34,35^. MoCA subscores could accelerate the diagnosis of dementia types, especially in the primary clinical centers with no access to imaging and in the cases which imaging is not helpful. Cognitive rehabilitation in patients with dementia is a very useful tool to improve the quality of life in these patients. We suggest that MoCA subscores could be useful to determine the field of rehabilitation in each patient, so it could be helpful in the management of the patients. The MoCA subscores could also exhibit the rate of cognition deterioration. For example, MoCA is a useful tool to precipitate cognition impairment in FTD patients^14^, and clock drawing test, attention, verbal fluency, and abstraction are indicators for progressive dementia in Parkinson's diseases^36^.

We also had some limitations in our study. Although the whole sample size of our study was relatively large, the sample size in each group was dropped because we included all patients during the time period, then divided them into groups, and did not include them based on a sample size calculation for each group. To investigate this prediction value, one should follow up the patients and compare MoCA results with worsening imaging and clinical features in different time periods.

In conclusion, using only the total MoCA score and subscores is not a valuable tool to differentiate types of dementia. MoCA subscores could give physicians some clues to put patients into dementia categories. MoCA subscores could benefit physicians in screening and categorizing patients into one dementia category, and it is useful alongside other imaging findings and clinical or paraclinical investigations.
